# Streamlined Analysis of Maternal Plasma Indicates Small Extracellular Vesicles are Significantly Elevated in Early-Onset Preeclampsia

**DOI:** 10.1007/s43032-024-01591-y

**Published:** 2024-05-22

**Authors:** Scout Bowman-Gibson, Chandni Chandiramani, Madison L. Stone, Christopher A. Waker, Traci M. Rackett, Rose A. Maxwell, David N. Dhanraj, Thomas L. Brown

**Affiliations:** 1 Department of Neuroscience, Cell Biology and Physiology, Boonshoft School of Medicine, Wright State University, 3640 Colonel Glenn Highway, 457 NEC Building, Dayton, OH 45435 USA; 2 Department of Obstetrics and Gynecology, Boonshoft School of Medicine, Wright State University, Dayton, OH 45435 USA

**Keywords:** Preeclampsia, Plasma, Extracellular vesicles, Exosomes, Hypoxia inducible factor 1 alpha

## Abstract

Preeclampsia (PE) is a leading cause of maternal and fetal mortality and morbidity. While placental dysfunction is a core underlying issue, the pathogenesis of this disorder is thought to differ between early-onset (EOPE) and late-onset (LOPE) subtypes. As recent reports suggest that small extracellular vesicles (sEVs) contribute to the development of PE, we have compared systemic sEV concentrations between normotensive, EOPE, and LOPE pregnancies. To circumvent lengthy isolation techniques and intermediate filtration steps, a streamlined approach was developed to evaluate circulating plasma sEVs from maternal plasma. Polymer-based precipitation and purification were used to isolate total systemic circulating maternal sEVs, free from bias toward specific surface marker expression or extensive subpurification. Immediate Nanoparticle Tracking Analysis (NTA) of freshly isolated sEV samples afforded a comprehensive analysis that can be completed within hours, avoiding confounding freeze–thaw effects of particle aggregation and degradation.

Rather than exosomal subpopulations, our findings indicate a significant elevation in the total number of circulating maternal sEVs in patients with EOPE. This streamlined approach also preserves sEV-bound protein and microRNA (miRNA) that can be used for potential biomarker analysis. This study is one of the first to demonstrate that maternal plasma sEVs harbor full-length hypoxia inducible factor 1 alpha (HIF-1α) protein, with EOPE sEVs carrying higher levels of HIF-1α compared to control sEVs. The detection of HIF-1α and its direct signaling partner microRNA-210 (miR-210) within systemic maternal sEVs lays the groundwork for identifying how sEV signaling contributes to the development of preeclampsia. When taken together, our quantitative and qualitative results provide compelling evidence to support the translational potential of streamlined sEV analysis for future use in the clinical management of patients with EOPE.

## Introduction

PE is a life threatening, pregnancy-specific condition that occurs in 4–7% of women worldwide [1–5]. Pathologic indications of PE are rapid, new onset hypertension after 20 weeks of gestation with accompanying proteinuria or maternal renal, pulmonary, or neurological involvement in the absence of proteinuria [6, 7]. This condition is one of the leading causes of maternal and fetal morbidity and mortality, and both preeclamptic mothers and their offspring are at higher risk of developing cardiovascular or metabolic diseases later in life [8–12]. The only cure for PE is delivery of the placenta [6, 7, 13]. No current treatment strategies or predictive measurements have proven to be successful in the clinical management of PE, emphasizing the substantial need to determine how this condition originates and how it can be identified.

At least two subtypes of PE are generally accepted to distinguish between patients diagnosed before 34 weeks of gestation (early-onset/EOPE) and patients diagnosed after 34 weeks of gestation (late-onset/LOPE) [6, 14–17]. A study by Benton et al., reported heterogenous placental profiles of PE using gene array [18]. However, a more recent report by He et al. using single cell sequencing analysis has indicated that when compared to LOPE, patients diagnosed with EOPE represent a more homogenous disease subtype primarily characterized by prolonged dysregulation of hypoxia-related signaling in the placenta after the first trimester [19]. Placental dysfunction as a result of reduced placental perfusion, oxidative stress, and/or hypoxia constitutes “compromised placental oxygen signaling” and likely acts as a primary driver of the pathogenesis of EOPE [19–23].

HIF-1α, a transcription factor essential for embryonic and placental development as well as fetal survival, is a critical molecular mediator of oxygen sensing. HIF-1α protein is significantly elevated in placental tissues after the first trimester in EOPE pregnancies [19, 22, 24–31]. EOPE placentas are reported to exhibit significantly higher levels of microRNA-210-3p (miR-210), a hypoxia-responsive microRNA which has been shown to form a positive signaling feedback loop with HIF-1α [32, 33]. Although inappropriate overactivation of hypoxia-related signaling in the placenta is a common feature of EOPE, it is not yet clear how placental HIF-1α may lead to maternal pathogenesis.

Maternal-placental communication normally occurs throughout gestation and is mediated in part by extracellular vesicles (EVs). EVs are lipid-bilayer nanoparticles that are secreted into circulation via numerous cell types and have been shown to be involved in intercellular communication by carrying biological cargo such as proteins, mRNA, and miRNA [34–44]. Although a multitude of distinct EV subpopulations have been identified, current research indicates that small extracellular vesicles (sEVs) play a central role in normal and pathological cell-to-cell communication [44–48]. sEVs are less than 200nm in size and include endocytic exosome (50-150nm) and plasma membrane microvesicle (100-200nm) subtypes [39, 43, 48–50]. Plasma sEV concentration has been shown to increase physiologically as a healthy pregnancy progresses, partly due to the growing placenta continually shedding vesicles into the maternal circulation [23, 40, 51, 52]. However, dysregulated sEV signaling has been reported to contribute to the pathogenesis of pregnancy-related disorders including gestational diabetes and PE [41, 48, 53–57].

Numerous methods of extracellular vesicle isolation and analysis have been utilized to further investigate the associations between sEV dysregulation and PE; however, sEVs are diverse in nature and no bona fide surface markers have been identified to differentiate between exosomal sEV and microvesicular sEV subpopulations [50, 58]. Many of the reported techniques for sEV analysis isolate specific subpopulations or require expensive instrumentation, large plasma volume input, and lengthy isolation steps that make it challenging to evaluate sEV data in a clinically useful manner [34–36]. Pregnant patient sEV analysis can vary significantly depending on the specific method of isolation and the intended readout and use of that information.

To circumvent sEV isolation techniques which have restrictive time, labor, or technological limitations, we have identified a streamlined approach to evaluate the total circulating concentration of maternal plasma sEVs in pregnancies complicated by early and late onset preeclampsia. This streamlined approach bypasses lengthy ultracentrifugation and size-exclusion procedures by employing polymer-based precipitation and subsequent bipartite resin column purification to rapidly produce high-yield sEV samples. Our technique eliminates excess filtration and surface-marker based sorting steps, giving a novel comprehensive representation of total systemic maternal sEVs that is unbiased towards sEV subpopulations. In addition, cargo held by sEVs isolated using this streamlined approach can be analyzed to assess the functional activity and predictive power of specific biomarkers relevant to the pathogenesis of PE. Using freshly isolated sEVs further avoids particle aggregation and degradation caused by storage conditions or repeated freeze-thawing [59]. Importantly, we believe this approach can be used to benefit PE patient management when applied in relevant clinical situations.

## Methods

### Study Groups and Samples

The population under study consisted of mothers aged 18–40 with singleton pregnancies: 14 healthy pregnant patients (Control) and 12 PE patients, with all donating samples between 24 and 39 weeks of gestation. 8 PE patients were identified as early-onset preeclamptic (EOPE) due to symptom onset prior to 34 weeks of gestation. 4 PE patients were identified as late-onset preeclamptic (LOPE) due to symptom onset after 34 weeks of gestation. While the gestational age at sample collection was not statistically different between study groups, the gestational age at delivery did differ (p < 0.001) (Table 1).

**Table 1:** Demographic and clinical patient information.

	Control (n=14)	EOPE (n=8)	LOPE (n=4)
Maternal Age	27.1 ± 1.6	26.6 ± 1.6	30.0 ± 3.2
Prepregnant Maternal BMI^+^	29.8 ± 1.8	36.2 ± 4.4	31.6 ± 2.4
Maternal Race			
African American	4 (28.5%)	0	0
Caucasian	9 (64.3%)	7 (87.5%)	4 (100%)
Hispanic	1 (7.1%)	1 (12.5%)	0
Maternal Blood Pressure (Systolic, mmHg)	113 ± 2	159^*^± 12	150* ± 5
Maternal Blood Pressure (Diastolic, mmHg)	68 ± 2	92^*^ ± 6	86* ± 3
Proteinuria^++^	0/12	4/5	3/4
Gestational age at sample collection (weeks)	31.0 ± 1.2	30.3 ± 1.2	29.9 ± 2.6
Gestational age at PE diagnosis (weeks)	N/A	29.9 ± 1.1	36.6 ± 0.9
Gestational age at delivery (weeks) Birth Weight (g)	39.5 ± 0.2 3525.0 ± 110.9	33.1* ± 0.9 1974.0* ± 254.6	37.6* ± 0.5 3275.0 ± 207.6

Data are reported as mean ± SEM, as percentages of the study population, or as individual parts of the study population. * indicates p < 0.001 versus Control when evaluated by unpaired t-test. + Prepregnant maternal BMI data was unavailable for 2 EOPE patients. + + Urinary protein was unavailable for 2 Control patients and 3 EOPE patients

All subjects were informed and gave written consent prior to their participation in this study at Miami Valley Hospital, a high-risk maternity referral center in Dayton, Ohio USA. This study was reviewed and approved by the Wright State University Institutional Review Board. Patients with autoimmune diseases, chronic hypertension, recurrent miscarriages, preterm premature rupture of membranes (PPROM), or Hemolysis, Elevated Liver enzymes and Low Platelets (HELLP) syndrome were excluded from this study.

PE was diagnosed based on the International Society for the Study of Hypertension in Pregnancy (ISSHP) criteria and identified as new-onset hypertension with two repeated systolic blood pressure measurements ≥ 140 mmHg and/or diastolic blood pressure ≥ 90 mmHg with coinciding proteinuria occurring after 20 weeks of gestation in previously normotensive patients or with indications of maternal renal, hepatic, or neurological involvement in the absence of proteinuria [60] (Table 1).

### Plasma Collection

Blood was drawn in K2/EDTA tubes, mixed well by inversion, placed on ice and centrifuged within 5 min at 1,500g for 10 min at room temperature to obtain platelet-rich plasma. After a subsequent centrifugation at 3,000g for 10 min, platelet-free plasma was aliquoted and stored at -80°C until sEV isolation and analysis. Plasma with visible hemolysis was excluded from analysis.

### sEV Isolation

**Fig. 1:**
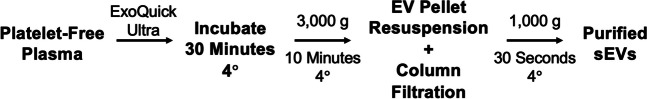
Streamlined sEV isolation and purification from maternal plasma. Whole blood drawn in K2/EDTA tubes was sequentially centrifuged to obtain platelet-free plasma prior to incubation with ExoQuick Ultra precipitation solution. All centrifugation steps were performed at 4ºC. Sizing and concentration analyses were performed immediately following sEV purification

Isolation, purification, and analysis of sEVs was performed in accordance with the guidelines recommended by the International Society for Extracellular Vesicles (ISEV) [39]. sEVs were isolated from platelet-free maternal plasma using ExoQuick Ultra (Systems Biosciences, #EQULTRA-20A-1) according to the manufacturer’s directions. Briefly, after centrifugation for 30 min at 14,500g to remove cellular debris, maternal plasma was combined with precipitation solution and incubated for 30 min at 4°C. Following incubation, samples were centrifuged for 10 min at 3,000g to obtain EV pellets. EV pellets were resuspended and loaded onto purification columns. A final centrifugation at 1,000g for 30 s yielded purified sEV samples (Fig. 1). Purified sEVs were immediately diluted for Nanosight analysis or stored at -80°C for downstream applications.

### Nanoparticle Tracking Analysis (NTA)

Extracellular vesicle size and concentration were evaluated using a Nanosight NS300 (Malvern Panalytical Instruments, UK) equipped with a 488 nm laser. NTA 3.4 software version was used to record and analyze sample videos. NTA tracking analysis measures the rate of Brownian motion of nanoparticles using light scattering to provide a reproducible and specific analysis of nanoparticle size and concentration [61]. Phosphate buffered saline (1X PBS) was twice filtered using 0.02 μm Anotop 25 syringe filters (Whatman, #6809–2102) and used to dilute plasma-derived sEVs. Measurements were performed with filtered PBS to confirm the absence of background particles. Samples were diluted in order to achieve 20–100 particles/frame. Final data were averaged from three recorded 30-s capture videos taken for each patient’s sEV sample. All capture videos were analyzed with detection threshold 3 and camera level 10. A minimum of 300 tracks per video were taken and samples were analyzed in triplicate.

### Western Blotting

Purified sEV samples were incubated on ice in 10X RIPA buffer supplemented with protease inhibitor cocktail (Cell Signaling Technology, #9806) and proteosome inhibitor MG-132 (Sigma, #M7449). Either 4X reducing sample buffer or 4X LDS non-reducing sample buffer (Thermo Scientific, #84,788) was added to the sEVs before heating at 95°C for five minutes. sEV samples were electrophoresed on SDS–polyacrylamide gels and subsequently transferred to a methanol-activated Immobilon-P Transfer Membrane (#IPVH00010 Immobilon). After blocking at room temperature for one hour with blocking buffer (1X TBS pH 7.6 containing 0.05% Tween-20 and 5% fat-free dry milk), membranes were incubated at 4°C with rocking overnight using primary antibodies for the following proteins: ALIX (1/1,000, Proteintech #67,715–1-Ig), β-Actin (1/10,000, Santa Cruz Biotechnology, #sc-47778), CD-9 (1/1,000, Proteintech #20,597–1-AP), Flotillin 1 (1/1,000, Proteintech #15,571–1-AP), GAPDH (1/2,000, Cell Signaling Technology #5174), Hif-1α (1/1,000, Novus Biologicals #NB100-449), placental alkaline phosphatase (1/1,000, Boster Biological Technology #A01718), TSG101 (1/1,000, Proteintech #28,283–1-AP). Following incubation, membranes were subsequently washed and incubated with secondary antibody at 1/10,000 for 1 h at room temperature and visualized via West Pico SuperSignal chemiluminescence.

### sEV miRNA Isolation and Analysis

Extracellular vesicle miRNA was isolated by Trizol-chloroform separation and silica spin-column extraction using 200ul of isolated plasma sEVs and miRNeasy Serum/Plasma Kit (Qiagen, #217,184) following the manufacturer’s instructions. RNA was eluted from the silica spin-column in nuclease free water. RNA concentration and purity was assessed by UV–Vis using NanoDrop One (Thermo Scientific). MiRNA-210-3P cDNA was synthesized using 3ul of isolated EV RNA, 3ul of the stem-loop RT primer from the Hsa-miRNA-210-3P qPCR Assay (ThermoFisher, #000512, PN: 4,427,975), and Taqman MicroRNA RT kit (ThermoFisher, #4,366,596). The resulting mixture was placed in a thermal cycler and incubated at 16°C for 30 min, 42°C for 30 min, and 85°C for 5 min. The resulting miRNA-210-3P cDNA was stored at 4°C until PCR amplification. MicroRNA cDNA synthesis was performed in conjunction with a no-template and a no-RT control. PCR amplification of isolated EV miRNA-210-3P was performed using cDNA, qPCR assay primers from the Taqman Hsa-miRNA-210-3P assay, and PCR Master Mix Fast Advanced (ThermoFisher, #4,444,556). The reaction mixtures were thermal cycled 35 times with a 5 s denature step at 95 °C and an annealing and extension period for 30 s at 60 °C. The resulting product was electrophoresed on a 4% w/v agarose gel containing ethidium bromide for 90 min. The presence of the approximately 50 bp product signified the presence of miRNA-210-3P in human plasma-derived sEVs. The absence of a band in the no-template and no-RT controls signified the amplification was due to the presence of the miRNA in isolated EVs and not the result of off-target amplification.

### Statistics

All data are reported as mean ± standard error of mean (SEM). GraphPad Prism 10 software was used for statistical analysis. Significance was determined between two group means by unpaired t-test, or between multiple group means by 1-way analysis of variance (ANOVA) with post hoc Bonferroni correction test to evaluate for pairwise comparisons. A p-value < 0.05 was considered statistically significant.

## Results

### Quantitative sEV Characterization

**Fig. 2:**
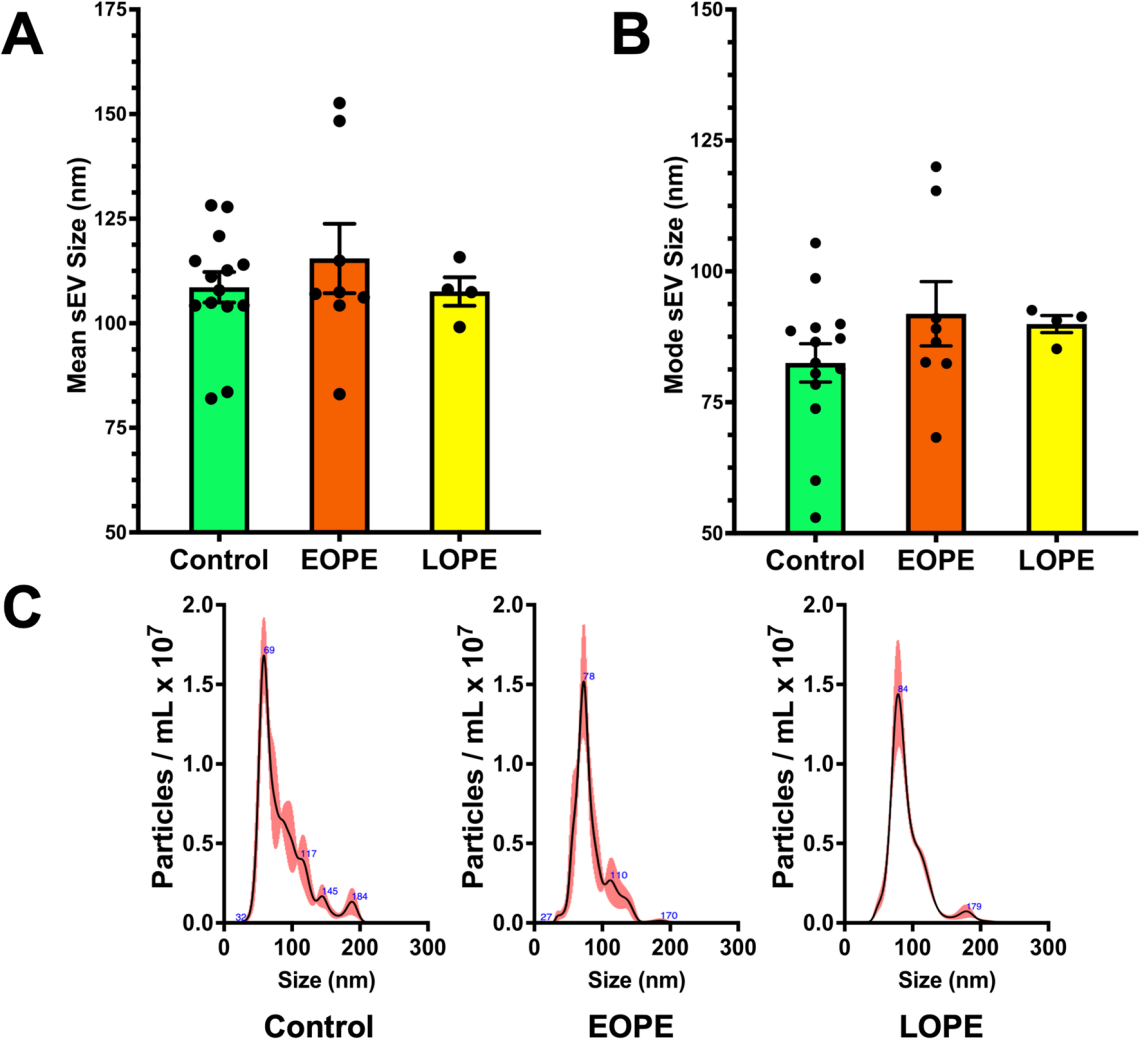
NTA for sEV size characterization. NTA was performed to characterize the sizing of sEVs isolated from the plasma of Control, EOPE, and LOPE patients. The mean diameter (**A**) and mode diameter (**B**) of Control, EOPE, and LOPE sEVs indicate that streamlined processing of maternal plasma produces sEV samples that fall within sizing range of sEV classification. There was no significant variation in mean or mode diameters between Control, EOPE, and LOPE sEVs. Representative graphs from individual patient NTA reports (**C**) indicate that similar particle size distributions were analyzed for each study population. All measurements are reported in nanometers (nm)

A streamlined approach was taken to rapidly isolate and analyze purified sEVs from Control, EOPE, and LOPE patient plasma. NTA indicated no significant differences in the average size of plasma sEVs from Control (108.6nm ± 3.6nm), EOPE (115.5nm ± 8.3nm), and LOPE (107.6nm ± 3.4nm) patients (Fig. 2A). The mode sizes of Control (82.5 ± 3.7nm), EOPE (91.9nm ± 6.1nm), and LOPE (90.0nm ± 1.6nm) sEVs also showed no significant variation (Fig. 2B). In addition to the mean and mode size measurements, representative particle size distribution graphs produced by NTA for individual patients from each study population indicates that our streamlined method reliably isolates sEVs less than 200nm and that these results are similar between PE and normotensive pregnancies (Fig. 2C).

### Qualitative sEV Characterization

**Fig. 3:**
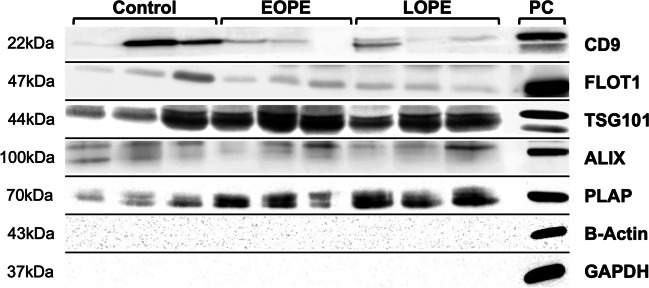
Western blot analysis of sEVs collected from Control, EOPE, and LOPE patients for markers indicating sEV biogenesis and placental tissue origin. Following sample lysis, sEV-bound proteins were electrophoresed on SDS-PAGE gels and analyzed for markers of EV biogenesis. The cytoskeletal protein Beta-actin and the glycolytic enzyme GAPDH were absent from all sEV samples. PC indicates the following positive controls: human term placental tissue (CD9, PLAP); HEK293 cell lysate (FLOT1, ALIX); Jurkat cell lysate (TSG101); Cos-7 cell lysate (B-Actin, GAPDH)

In addition to NTA size analysis, Western blotting was used to confirm sEV isolation from maternal plasma. Purified sEVs from Control, EOPE, and LOPE patients contained protein markers indicative of exosome biogenesis including the membrane-associated scaffolding protein flotillin-1, the tetraspanin protein CD9, and the endosomal accessory protein ALIX (Fig. 3). TSG101, a major component of the endosomal sorting complex required for transport (ESCRT) that commonly signifies exosomal and microvesicular sEV subpopulations, was present within control, EOPE and LOPE sEV samples (Fig. 3). Control, EOPE, and LOPE sEVs were negative for cytosolic housekeeping proteins B-actin and glyceraldehyde-3-phosphate dehydrogenase (Fig. 3). Placental alkaline phosphatase protein (PLAP), a marker of placental-derived extracellular vesicles, was detected within Control, EOPE, and LOPE plasma sEVs (Fig. 3) [53]. The presence of these markers provides confirmation that sEVs isolated from maternal plasma include exosomal subpopulations, as well as sEVs originating from placental tissue. Although Western blots were not quantified due to lack of a universal marker, sEV samples from EOPE and LOPE patients were highly enriched in PLAP and TSG101 when compared to Control plasma sEVs (Fig. 3).

### Maternal Plasma sEV Concentration Analysis

**Fig. 4:**
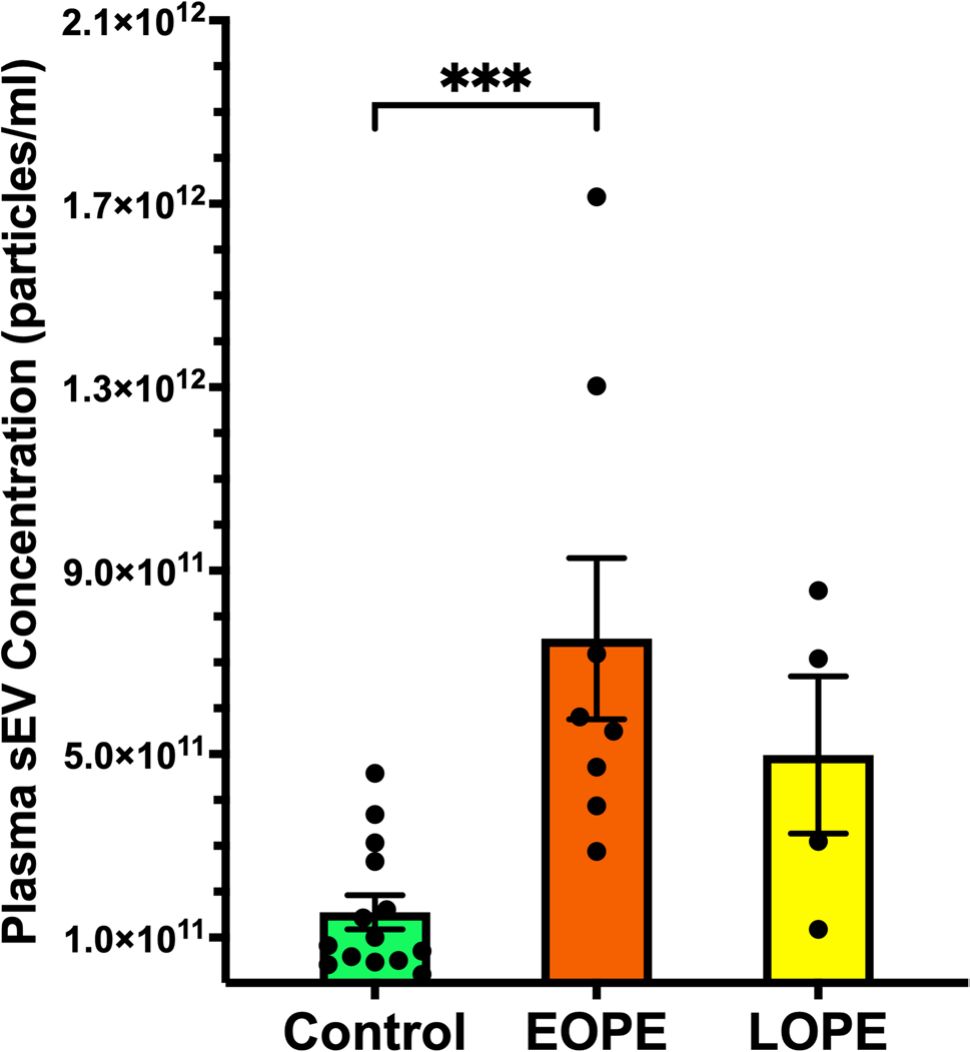
NTA for Quantification of maternal plasma sEVs from normotensive and PE pregnancies. When compared to Control, EOPE patient sEV concentrations were significantly increased (***p = 0.0010). LOPE patient sEVs were not significantly elevated over Control sEVs (p = 0.6187). All sEV concentration values are reported as particles per milliliter of plasma

NTA analysis revealed that sEVs from EOPE patients were significantly elevated when compared to normotensive pregnancies. Our data indicate that the concentration of sEVs in EOPE pregnancies (7.5 × 10^11^ ± 1.8 × 10^11^ particles/ml) is 5 times the amount present in Control pregnancies (1.5 × 10^11^ ± 3.7 × 10^10^ particles/ml, p = 0.0010, Fig. 4). There was no statistically significant difference between LOPE and normotensive pregnancies (Fig. 4). In addition, no significant differences were present when LOPE was compared to EOPE (p = 0.6187, Fig. 4).

### Detection of HIF-1α and miR-210 in sEVs

**Fig. 5:**
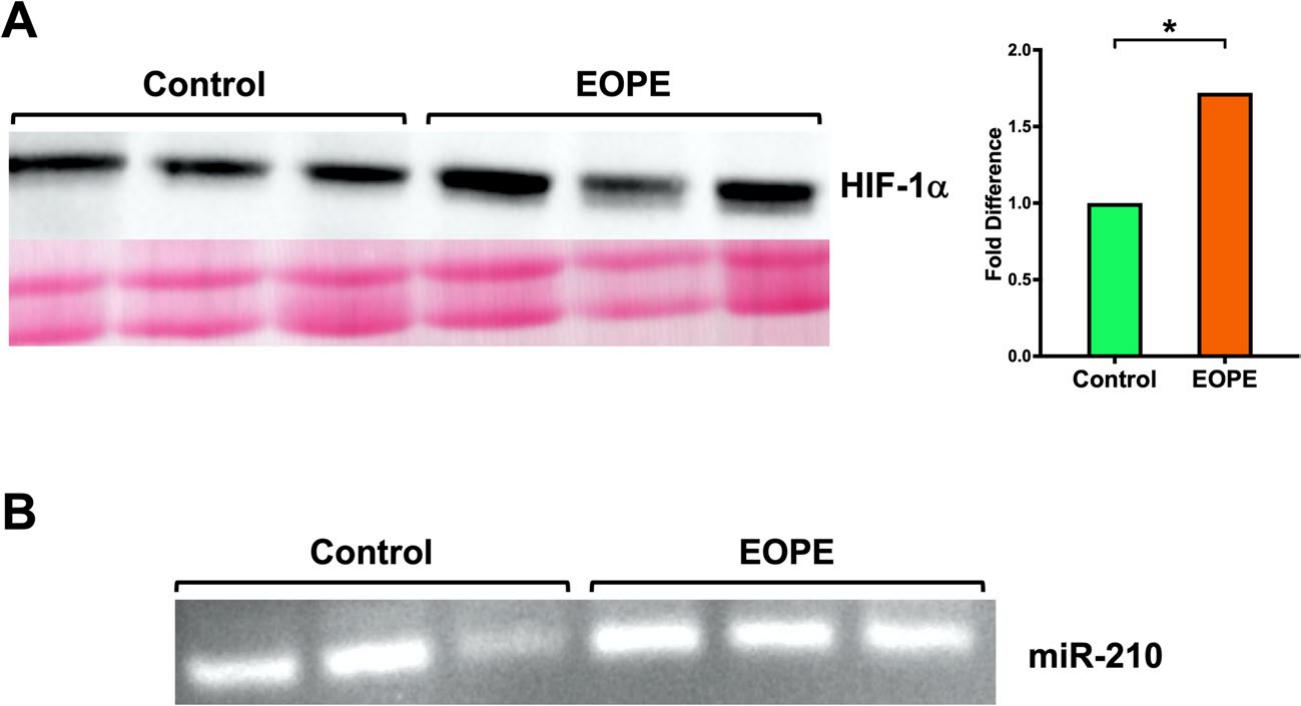
Western blot and miRNA analysis of sEVs from Control and EOPE pregnancies. HIF-1α, a master regulator of oxygen sensing, and its directly inducible target miR-210 were both detected within Control and EOPE sEVs. Relative HIF-1α protein levels were found after normalizing bands to total protein after staining with 1% Ponceau S. EOPE sEVs were found to harbor significantly more HIF-1α than Control sEVs (* p = 0.0170) (**A**). miR-210 was also detected in both EOPE and normotensive study populations (**B**)

Upregulation of both HIF-1α and miR-210 has been implicated in oxidatively-compromised placentas and is thought to underly EOPE pathogenesis [2, 22, 24–28, 30, 31, 33, 62–66]. It is largely unknown whether excessive HIF-1α protein in preeclamptic placentas could bypass degradation and travel systemically to transcriptionally modify other tissues in normoxia. As our results indicated that patients with EOPE have significantly more plasma sEVs in circulation than normotensive pregnancies, we wanted to determine whether plasma sEVs could serve as a potential transport system for HIF-1α protein or its target substrates. sEV lysis and subsequent Western blotting revealed that stabilized full length HIF-1α protein is present within EOPE and Control sEVs (Fig. 5A). EOPE patient sEVs had significantly higher levels of HIF-1α protein than Control patient sEVs (p = 0.0170, Fig. 5A).

HIF-1α signaling is known to induce production of miR-210, and placental miR-210 upregulation has also been extensively linked with EOPE pathogenicity [32, 33, 66–76]. To further explore the potential for sEVs to participate in HIF-1α-mediated signaling, we extracted sEV-bound small RNAs to then determine if hypoxia-inducible miR-210 was also located within maternal plasma sEVs. Our data indicate that miR-210 was detected in plasma sEVs from Control and EOPE pregnancies (Fig. 5B).

## Discussion

EVs are lipid bilayer-enclosed structures produced from endocytic formation or from direct cellular plasma membrane blebbing. Upon their release into circulation, EVs are capable of carrying a wide variety of cargo capable of participating in cellular signaling [37, 42–47, 58, 77, 78]. There is now widespread recognition that EVs less than 200 nm in size, defined as sEVs, significantly contribute to cell-to-cell communication through the delivery of bioactive contents to downstream target tissues [42, 43, 78–80].

In the setting of human pregnancy, plasma EV concentrations are known to increase physiologically [48, 52, 81–84]. As a healthy pregnancy progresses, the placenta releases EVs into circulation as a means of communicating with maternal organs and promoting the various physiologic adaptations necessary to support the growing fetus [48, 51, 52, 85]. In vitro studies support advantageous sEV signaling in pregnancy: placental sEVs may be beneficially immunomodulatory and may function to enhance maternal spiral artery remodeling [77, 81, 84]. Conversely, there is now also wide-spread evidence demonstrating the pathologic impact of sEV signaling in the pathogenesis of pregnancy-related disorders including gestational diabetes and PE [23, 41, 42, 54, 55, 78–80, 86–92].

PE is pregnancy-specific de novo hypertension with concomitant signs of maternal organ involvement occurring after 20 weeks of gestation; the early onset subtype of PE occurring before 34 weeks of gestation is thought to have different underlying issues than those associated with the late onset subtype of PE diagnosed after 34 weeks of gestation [6, 14, 17, 19, 22, 60]. LOPE occurs in approximately 70% of PE patients and generally is associated with normal placental development [15, 20, 93]. EOPE makes up the most severe cases occurring in 15–20% of PE patients and is strongly associated with abnormal placental development and compromised placental oxygen signaling [2, 6, 14, 15, 60, 94].

Regardless of onset subtype, preeclamptic placentas are believed to communicate host organ damage to distant maternal tissues through modified sEV production and release [51, 87, 88, 90, 95]. The dynamic release of sEVs in reaction to maternal organ dysfunction, along with the stability of sEVs and their cargo within biological fluids such as plasma, has brought attention to utilizing noninvasive liquid biopsies and sEV characterization for diagnosing or monitoring the progression of pregnancy-related disorders including PE [37, 53, 80, 88, 90]. Specific sEV subpopulations, such as PLAP + syncytiotrophoblast-derived microparticles (STBMs) and CD68 + exosomes, have been reported to be elevated in PE patient plasma [23, 53, 86, 96–100]. However, isolating and quantifying sEV subpopulations is often labor-and time-intensive making it difficult to implement in a clinical setting.

As a reproducible approach to sEV analysis, we opted to streamline the isolation and quantification of plasma sEVs from normotensive and preeclamptic pregnancies. With minimal volume input and greatly reduced sample handling time, we have identified that EOPE pregnancies are associated with a significant increase in maternal plasma sEVs. The concentration values reported in this study represent measurements of circulating sEVs smaller than 200nm as detected by NTA, accounting for overlapping sEV subpopulations. While our approach to sEV isolation was conducted independent of surface protein expression, our results from Western blot confirm that various markers associated with exosomal, microvesicular, and placental-derived EV subtypes were present in our samples after purification. Aside from previously reported increases in particular sEV subpopulations, the significant increase in total plasma sEV concentrations we have identified in EOPE patients could suggest that the early onset PE phenotype may also be associated with systemically augmented maternal end organ sEV production.

The variable significance between EOPE and LOPE sEV concentrations versus Control sEV concentrations that we report are consistent with distinct pathogeneses of each PE subtype. LOPE patients generally have normal placentation, but excessive fetal metabolic demand late in the third trimester is thought to overwhelm the functional capacity of the placenta and cause placental cell stress without evidence of abnormal maternal perfusion [15, 23, 93]. In contrast, abnormal placentation and reduced perfusion due to compromised placental oxygen signaling are thought to contribute to the pathogenesis that primarily characterizes early onset PE phenotypes [19, 22, 30, 55, 93, 96]. Shallow decidual invasion and incomplete spiral artery remodeling are key pathogenic features underlying EOPE, and EOPE placentas commonly exhibit signs of oxidative placental cell stress [10, 15, 22, 25, 27, 28, 30, 62].

Numerous studies have consistently reported that HIF-1α protein, a master regulator of oxygen sensing, is significantly elevated in placental tissues after the first trimester in EOPE patients when compared to normotensive controls [22, 24–30, 63]. Although HIF-1α signaling is essential in the early stages of pregnancy, increased oxygenation at around 10 weeks of gestation normally leads to rapid HIF-1α protein degradation in the placenta; however, persistent stabilization and elevation of placental HIF-1α protein beyond the low-oxygen intrauterine environment of the first trimester is strongly associated with the pathogenesis of EOPE [2, 22, 24–31, 63, 64, 66]. Recent animal models have further reinforced this notion: trophoblast-specific HIF-1α overexpression, as well as conditional knockout of prolyl hydroxylase domain protein 2, result in pathologic HIF-1α stabilization and consequently induce EOPE-like symptoms in mice [2, 31].

In addition to elevated HIF-1α protein stabilization, EOPE is associated with abnormal expression of hypoxia-responsive miRNAs in the placenta [19, 24, 27, 33, 63]. Typically comprised of 22–25 nucleotides, miRNAs are small non-coding RNA molecules capable of translationally modifying gene expression through direct binding at the 3’-untranslated region (UTR) of mRNA [33, 69, 75, 101]. Although many placental miRNAs have been shown to be dysregulated in PE, abnormal upregulation of the hypoxia-responsive miR-210 in placental tissues has been particularly associated with EOPE [32, 33, 67, 69]. Hypoxic activation of miR-210 has been reported to occur via a HIF-1α-dependent mechanism [66]. miR-210 overexpression further induced HIF-1α protein stabilization, creating a positive feedback loop that exacerbated downstream activation of numerous hypoxia-related signaling pathways [33]. While pathologic upregulation of both miR-210 and a HIF-1α has been extensively implicated in the placental dysfunction that occurs in EOPE, it is not yet clear how the placenta communicates with other organ systems to relay compromised placental oxygen signaling and induce maternal pathophysiology [10, 19, 27, 32, 33, 71, 86, 102]**.**

However, studies conducted by Aga et al. revealed that human nasopharyngeal carcinoma and epithelial cell lines were able to package full-length HIF-1α in sEVS [103]. Furthermore, studies by Ferreira et al. demonstrated that full-length HIF-1α protein released from sEVs retained functionality independent of oxygen tension [103, 104]. These reports indicate that HIF-1α protein is protected from degradation when packaged within sEVs and that sEV-bound HIF-1α is functionally capable of modifying its downstream signaling when taken up by target cells in normoxia [103, 104]. Biró et al. further detected the presence of miRNA-210 in an exosomal CD63 + subpopulation [71]. When considering the placenta’s continuous release of EVs throughout pregnancy, the characteristic elevation of placental HIF-1α protein and miR-210 seen in EOPE placentas may lead to preferential packaging of these molecules into sEVs for secretion into maternal circulation. Upon maternal tissue uptake, intracellular release of sEV-bound HIF-1α and miRNA-210 could initiate inappropriate hypoxia-related signaling and further dysregulate systemic sEV secretion. While further investigation is required to validate the pathogenicity of sEV-bound HIF-1α and miR-210, our studies represent a promising first step in elucidating the molecular origins of EOPE.

Our studies are the first to report that full-length HIF-1α protein is present in maternal plasma sEVs during pregnancy. Moreover, we found that HIF-1α protein was significantly elevated in sEVs from patients with EOPE compared to normotensive pregnancies. In addition to HIF-1α, we have shown its downstream target, miR-210, is also encapsulated and present systemically in maternal sEVs. These results suggest that sEVs structurally protect HIF-1α and miR-210 from systemic degradation.

In conclusion, we have utilized a rapid and reliable streamlined approach to analyze maternal plasma from pregnant patients and have determined that sEVs are significantly elevated in the maternal plasma of EOPE patients and contain HIF-1α and its direct target miR-210. The rapidity and reliability of sEV quantification and characterization offered by this approach is a significant advance when working with pregnancy-related disorders such as PE, as it can provide a greater potential for detection leading to earlier medical interventions to prolong pregnancy and minimize preterm birth-related complications.

Our study has some limitations in that plasma was collected from a small sample size and analyzed at a single gestational time point. Although these limitations exist, findings from this study provide compelling evidence that our streamlined approach to comprehensively assess maternal sEVs from minimally invasive liquid biopsies can distinguish a significant sEV elevation in EOPE pregnancies when compared to normotensive pregnancies. These results open the door for future studies with expanded sample sizes and longitudinal sEV analysis to aid in the clinical management of EOPE. This report provides novel insight into the potential genesis of EOPE: as the presence of full-length HIF-1α protein in conjunction with miR210 in circulating maternal sEVs may explain how compromised placental oxygen signaling may lead to pathophysiologic responses in downstream maternal tissues.

## Data Availability

All data and material are available upon request.
